# Synthetic Isoliquiritigenin Inhibits Human Tongue Squamous Carcinoma Cells through Its Antioxidant Mechanism

**DOI:** 10.1155/2017/1379430

**Published:** 2017-01-22

**Authors:** Cuilan Hou, Wenguang Li, Zengyou Li, Jing Gao, Zhenjie Chen, Xiqiong Zhao, Yaya Yang, Xiaoyu Zhang, Yong Song

**Affiliations:** ^1^School of Basic Medical Sciences, Lanzhou University, Lanzhou, Gansu Province 730000, China; ^2^Key Lab of Preclinical Study for New Drugs of Gansu Province, Lanzhou, Gansu Province 730000, China; ^3^Department of Physiology and Pathophysiology, Fudan University Shanghai Medical College, Shanghai 200000, China; ^4^Jining Medical University, Jining, Shandong Province 272067, China; ^5^Northwest Nationalities University Hospital, Lanzhou, Gansu Province 730000, China; ^6^School of Public Health, Curtin University, Centre for Genetic Origins of Health and Disease, The University of Western Australia and Curtin University, M409, 35 Stirling Highway, Crawley, WA 6009, Australia

## Abstract

Isoliquiritigenin (ISL), a natural antioxidant, has antitumor activity in different types of cancer cells. However the antitumor effect of ISL on human tongue squamous carcinoma cells (TSCC) is not clear. Here we aimed to investigate the effects of synthetic isoliquiritigenin (S-ISL) on TSCC and elucidate the underlying mechanisms. S-ISL was synthesized and elucidated from its nuclear magnetic resonance spectrum and examined using high performance liquid chromatography. The effects of S-ISL on TSCC cells (Tca8113) were evaluated in relation to cell proliferation, apoptosis and adhesion, migration, and invasion using sulforhodamine B assay, fluorescence microscopy technique, flow cytometry (FCM) analysis, and Boyden chamber assay. The associated regulatory mechanisms were examined using FCM and fluorescence microscopy for intracellular reactive oxygen species (ROS) generation, Gelatin zymography assay for matrix metalloproteinase (MMP) activities, and Western blot for apoptosis regulatory proteins (Bcl-2 and Bax). Our data indicated that S-ISL inhibited Tca8113 cell proliferation, adhesion, migration, and invasion while promoting the cell apoptosis. Such effects were accompanied by downregulation of Bcl-2 and upregulation of Bax, reduction of MMP-2 and MMP-9 activities, and decreased ROS production. We conclude that S-ISL is a promising agent targeting TSCC through multiple anticancer effects, regulated by its antioxidant mechanism.

## 1. Introduction

Squamous cell carcinoma of the tongue (TSCC) is one of the most common malignant tumors in the oral cavity and accounted for approximately 30% of all oral cancers in the United States in 2006 [1]. Moreover, its incidence has increased over the past decades worldwide [2]. Despite advances in chemotherapy, radiotherapy, and surgical therapy, the clinical outcomes and overall survival rates of TSCC have not been significantly improved over the last decades with overall five-year survival rate of less than 50% [3]. The high morbidity and mortality of oral cancers are largely due to rapid tumor growth, frequent tumor recurrence, and metastasis. Therefore, it is important to identify and develop novel agents which could simultaneously target abnormal proliferation, apoptosis, invasion, and metastasis of tongue cancer.

Isoliquiritigenin (ISL), 2′, 4′, 4′-three hydroxychalcone (molecular structure shown in Supplementary Figure  a in Supplementary Material available online at https://doi.org/10.1155/2017/1379430), mainly presents in roots of licorice and many other plants, foods, beverages, and tobaccos [4]. ISL possesses a wide variety of potent biological and pharmacological activities, including anti-inflammatory [5], antivirus [6], antioxidative [5], antiaging [7], and antidiabetic activities [8]. We previously showed that ISL could significantly reduce cardiac reactive oxygen species (ROS) level during hypoxia/reoxygenation, rendering protection against myocardial ischemic injury [9] and inhibiting the growth of prostate cancer cells [10]. ISL is reported to have anticarcinogenic effects in both in vivo and in vitro experimental models. In vivo studies revealed that ISL inhibited chemically induced colonic tumorigenesis [11], skin papilloma formation [12], and lung metastasis of murine renal carcinoma cells [13]. In vitro studies showed that ISL had antiproliferation activities in skin [14], pulmonary [13], breast [15], prostate [10], and gastric cancer cells [16]. A recent study showed that ISL induced human oral squamous cell carcinoma cell cycle G_2_/M phase arrest, apoptosis, and DNA damage [17], implying that ISL is a promising chemopreventive agent against oral cancer. However the antitumor effect of ISL on TSCC is not fully characterized. In the present study, we aimed to further investigate antiproliferative, proapoptotic, and antimetastatic effects of ISL on human tongue squamous carcinoma cells and elucidate the underlying mechanisms. Since natural ISL compound preparation is expensive with poor extraction rates and particularly wastes or destroys natural resources, we selected to observe antitumor effects of chemically synthesized ISL (S-ISL) in the study, which has great advantages in future preclinical development and clinical use, for example, reducing production costs and protecting licorice natural resources.

## 2. Materials and Methods

### 2.1. The Synthesis of S-ISL

S-ISL was synthesized and elucidated from its nuclear magnetic resonance spectrum (Supplementary Figure) as previously described [18]. The mixture of ethanol (5.6 mL), 2, 4-dihydroxyacetophenone (1, 6.8 g, 44.7 mmol) and 4-hydroxybenzaldehyde (2, 5.6 g, 45.9 mmol) was added to aqueous potassium hydroxide (41.6 mL, 60% w/w). The above suspension was heated at 100°C for 1.5 h and then stored overnight at room temperature. The reaction mixture was poured onto ice (100 g) and acidified to pH 4 using cold hydrochloric acid. The precipitated yellow solid was filtered, washed with water (200 mL), and air-dried to a yellow solid (3, 7.5 g, 65%). ^1^H NMR (400 MHz, (CD_3_) CO): *δ* 6.37 (s, 1 H), 6.47 (d,* J* = 8.0 Hz, 1 H), 6.93 (d,* J* = 8.0 Hz, 2 H), 7.74~7.86 (m, 4 H), 8.13 (d,* J* = 8.0 Hz, 1 H), 9.00 (s, 1 H), 9.47 (s, 1 H), 13.65 (s, 1 H); ^13^C NMR (100 MHz, (CD_3_) CO): *δ* 103.85, 108.76, 114.61, 116.86, 118.37, 127.67, 131.88, 133.38, 145.24, 161.07, 165.61, 167.67, 192.93 (Supplementary Figures b and c). Finally the purity of S-ISL was analyzed by high performance liquid chromatography (HPLC) method [19] with a C_18_ column (5 *μ*m, 4.6 × 250 mm), mixture of methanol and water (80 : 20, v : v) with rate of 1.0 mL/min, and detected at 370 nm. Each sample solution (10 *μ*L) was injected into the analysis system (Supplementary Figure e). The purity of S-ISL acquired was more than 95% and used in the subsequent studies.

### 2.2. Cell Culture

For comparison of specificity and sensitivity of S-ISL in carcinoma cells, we initially screened human tongue squamous carcinoma Tca8113 cells, human liver carcinoma HepG2 cells, and rat pheochromocytoma PC12 cells obtained from Chinese Academy of Sciences (Shanghai, China). The cells were grown in complete Dulbecco's modified Eagle's medium (DMEM; Invitrogen, Gibco, Grand Island, NY, USA) and supplemented with 10% heat-inactivated fetal bovine serum at 37°C in a humidified atmosphere containing 95% air/5% CO_2_. Exponentially growing cells were used for experiments. S-ISL was dissolved in dimethylsulfoxide (DMSO) to make a 10 mg/mL stock solution, which was further diluted to appropriate concentration with culture medium before each experiment.

### 2.3. Cell Viability Analysis Using Sulforhodamine B Assay

The effects of S-ISL on the viability of Tca8113, HepG2, and PC12 cells were determined using sulforhodamine B (SRB) assay (Sigma) [20]. Tca8113 cells (3.5 × 10^4^ cells/mL), HepG-2 cells (7 × 10^4^ cells/mL), and PC12 cells (7 × 10^4^ cells/mL) were seeded in 96-well plates and were separately treated with various concentrations of S-ISL for 24 h, 48 h, and 72 h. The optical density in each well was read using microplate reader at 570 nm. The experiment was repeated at least three times.

### 2.4. Determination of Intracellular ROS Generation

Intracellular ROS generation was evaluated using dichlorodihydrofluorescein diacetate (DCFH-DA) assay (Sigma, St Louis, MO, USA), which is a specific probe for hydrogen peroxide to form fluorescent dichlorofluorescein [9]. All groups were added with stimulant H_2_O_2_ (100 *μ*M) except the vehicle group (0.5% DMSO) prior to treatment with 10 *μ*M DCFH-DA at 37°C for 30 min. After incubation, cells were immediately submitted to fluorescence microscopy or flow cytometry and estimated using FL-1 channel.

### 2.5. Cell Cycle Analysis Using Flow Cytometry

Cell cycle analysis was carried out using flow cytometry (FCM) [21]. After Tca8113 cells were treated as described above, the cells were harvested and washed twice with ice-cold phosphate-buffered saline (PBS) and fixed with precooled ethanol (70% v/v) overnight. Subsequently the cells were stained with propidium iodide in PBS added with RNase in the dark at room temperature for 30 min. The sample was read on a Coulter Epics XL flow cytometry (Beckman-Coulter Inc, Fullerton, CA, USA).

### 2.6. Apoptosis Detection with Double Dye Annexin V-FITC/PI and 4′, 6-Diamidino-2-Phenylindole Dihydrochloride (DAPI) Staining

The double dye Annexin V-FITC/PI was used to distinguish between living cells, early and late apoptotic cells, and necrotic cells [22]. The cells were treated in the same manner as the cell cycle analysis, except no cooled 70% ethanol was added. For FCM analysis, the cells were stained with Annexin V-FITC/PI and detected by FCM using Annexin V-FITC cell Apoptosis Detection Kit (Sigma) according to manufacturer's instructions.

For fluorescence microscope examination, Tca8113 cells treated with S-ISL as described above were washed with ice-cold PBS twice, fixed with ethanol for 30 min, and stained with DAPI (0.1 *μ*g/mL, 2 min) at room temperature away from light. The stained cells were photographed with a fluorescence microscope (Olympus, Japan) at excitation wavelength of 480 nm and emission wavelength of 530 nm.

### 2.7. Western Blotting Analysis

The proteins in total cell lysates were separated on a 10% sodium dodecyl sulphate (SDS)-polyacrylamide gel electrophoresis and transferred onto polyvinylidene fluoride membrane (Millipore, Bedford, MA, USA). Membranes were blocked with 5% nonfat milk in tris-buffered saline buffer (pH 7.4) containing 0.1% Tween-20 for 1 h and subsequently incubated with primary antibodies (1 : 1000 dilution) against Bcl-2, Bax (Santa Cruz Biotechnology, Texas, USA) and GAPDH (Proteintech, Rosemont, USA) at 4°C overnight. Immunoreactive bands were detected using anti-rabbit horseradish peroxidase-conjugated secondary antibodies (1 : 3000 dilution) (Beyotime Biotechnology, Jiangsu, China) and visualized using LumiPico® ECL Reagent (Beyotime Biotechnology). The densities of immunoblotting bands were analyzed using a scanning densitometer (model GS-800; Bio-Rad, Shanghai, China) coupled with Bio-Rad personal computer analysis software.

### 2.8. Assays for Adhesion, Migration, and Invasion

Cell-matrix adhesion assay was carried out as described previously [23]. Briefly the 96-well plates were precoated with 0.04 *μ*g/*μ*L matrigel 50 *μ*L in triplicate overnight at 4°C and then washed with wash buffer (0.1% bovine serum albumin in medium). S-ISL pretreated cells were seeded in the precoated 96-well plates and incubated at 37°C. After 2 hours, nonadherent cells were washed away and attached cells were counted (Image-plus) and photographed from six randomly selected fields under an inverted microscope (Olympus, Beijing, China). The cell migration assay in vitro was performed by using a modified Boyden chamber (Millipore, Billerica, MA, USA) inserted with polyethylene terephthalate filter membrane containing 8 *μ*m pores in 24 well culture plates [24]. For cell migration assay, Tca8113 cells (2 × 10^5^ cells/mL) in serum-free medium, with or without S-ISL, were seeded in triplicate in the upper chamber. Complete medium was placed in the lower wells. The culture plates were incubated at 37°C in a 5% CO_2_ atmosphere. After incubation, the medium in the upper chamber was removed and washed with PBS twice. The cells remaining on the upper surface of the filter membrane were removed with cotton swabs and the cells on the opposite surface of the filter membrane were stained with 0.1% crystal violet for 5 min. The migrated cells were subjected to microscopic examination as described for the adhesion assay. For invasion assay, the whole process was the same as migration assay except that Tca8113 cells were loaded on presolidified matrigel.

### 2.9. Gelatin Zymography Assay

The activities of matrix metalloproteinase (MMP)-2 and MMP-9 in the conditioned medium were determined by Gelatin zymography assay [25]. Briefly, the serum-free medium was collected by centrifugation to remove cells and cell debris and subsequently loaded under nonreducing sample buffer onto SDS-polyacrylamide gel polymerized with 1% Gelatin (Sigma). Following electrophoresis, the gel was washed twice with rinsing buffer at room temperature for 1.5 h to remove SDS and then incubated in a developing buffer at 37°C overnight. Gels were stained with 0.1% Coomassie Brilliant Blue R-250 and destained in the same solution without dye. Gelatinase activity was visualized as clear bands against the blue-stained Gelatin background.

### 2.10. Statistical Analysis

Data were expressed as mean ± SD of multiple separate experiments. Statistical comparisons were performed using analysis of variance (ANOVA) followed by Student-Newman-Keuls' post hoc test for multiple comparisons using the computer statistical package (SPSS 21.0 for Window). Differences with *P* < 0.05 were considered statistically significant.

## 3. Results

### 3.1. Effects of S-ISL on Proliferation of Tca8113, HepG2, and PC12 Cells

**Figure 1:**
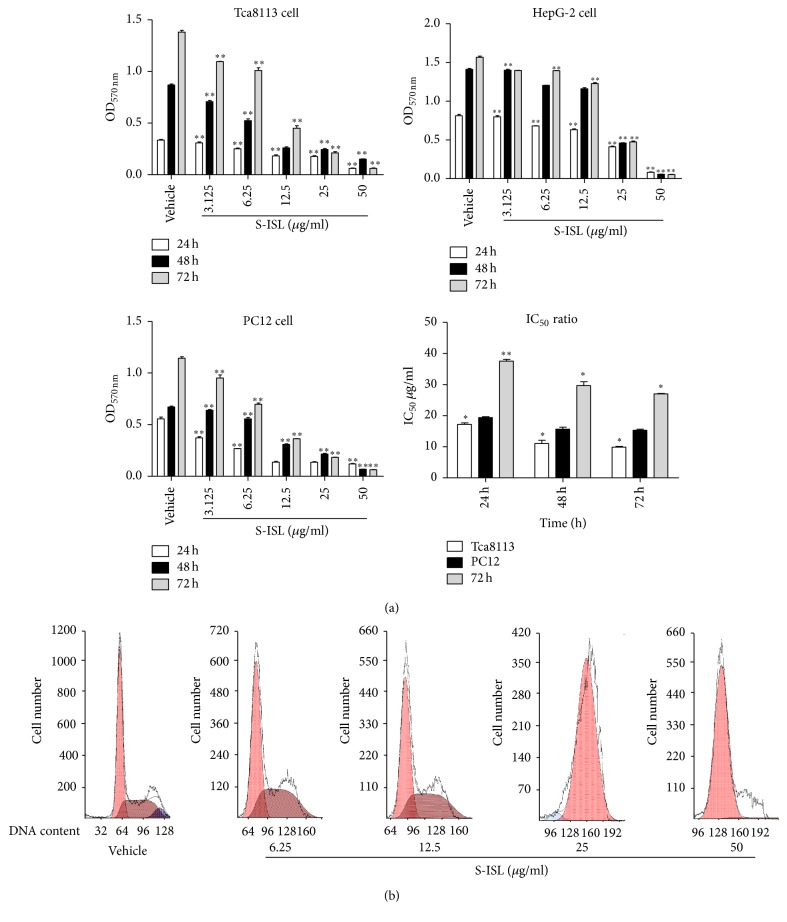
Effect of S-ISL on proliferation and cell cycle of cancer cells: The effects of S-ISL (3.125~50 *μ*g/mL) on Tca8113, HepG2 and PC12 cells were observed for 24 h, 48 h, and 72 h using SRB assay (a) and the effects of S-ISL (6.25~50 *μ*g/mL) on Tca8113 cell cycle were observed using FCM analysis (b). SRB results were expressed as the mean ± SD of three experiments with five replicates. S-ISL: synthetic isoliquiritigenin; SRB: sulforhodamine B; OD: optical density; IC_50_: 50% growth inhibition concentration; FCM: flow cytometry. ^*∗*^*P* < 0.05, ^*∗∗*^*P* < 0.01 versus the vehicle group.

The effects of S-ISL on the proliferation of Tca8113, HepG2, and PC12 cells were analyzed using SRB analysis. S-ISL markedly inhibited the proliferation of the above cells (Figure 1(a)), particularly Tca8113 cells and the IC_50_ values were 17.70 *μ*g/mL, 10.04 *μ*g/mL, and 9.67 *μ*g/mL after 24 h, 48 h, and 72 h treatment, respectively. HepG2 cells displayed intermediate responses to S-ISL and the IC_50_ values were 19.07 *μ*g/mL, 15.08 *μ*g/mL, and 14.95 *μ*g/mL, respectively, after the same treatment time. However, PC12 cell line was particularly resistant to S-ISL and the IC_50_ values were 38.13 *μ*g/mL, 30.94 *μ*g/mL, and 26.85 *μ*g/mL, respectively. As the present study demonstrated that Tca8113 were the most sensitive cells responding to S-ISL treatment, the subsequent studies were then focused on observing the effects of S-ISL on Tca8113.

### 3.2. Effects of S-ISL on Intracellular ROS Generation

**Figure 2:**
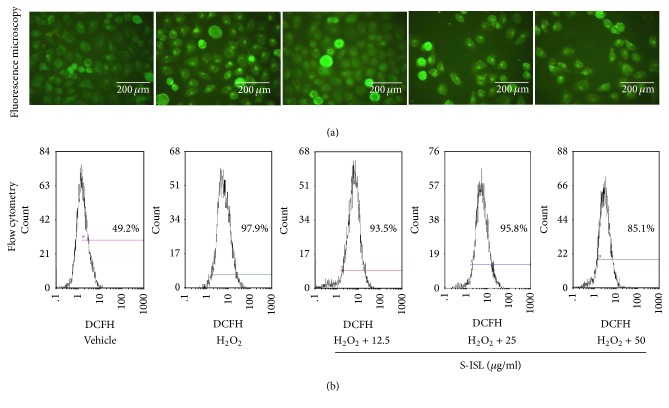
Effects of S-ISL on intracellular ROS levels: all groups were added with stimulant H_2_O_2_ (100 *μ*M) except the vehicle group. The change of ROS levels in response to S-ISL (12.5~50 *μ*g/mL) was examined using DCFH-DA analysis (a) and FCM analysis (b). S-ISL: synthetic isoliquiritigenin; ROS: reactive oxygen species; DCFH-DA: 2′, 7′- dichlorodihydrofluorescein diacetate; FCM: flow cytometry.

ROS production was measured in Tca8113 cells using DCFH-DA by fluorescence microscopy and FCM. As shown in Figure 2(a), when S-ISL was incubated with Tca8113 cells at varied concentrations from 12.5~50 *μ*g/mL, relative to H_2_O_2_ treatment alone, S-ISL groups reduced the fluorescence values by 2.7%, 9.3%, and 18.6%, respectively. Likewise, H_2_O_2_ increased ROS production by 48.7% as compared with the vehicle group under FCM analysis (Figure 2(b)). Compared with H_2_O_2_ treatment alone, pretreatment with S-ISL at concentrations ranging from 12.5~50 *μ*g/mL resulted in a decrease of intracellular ROS production and inhibition rate was 9.03%, 4.31%, and 26.22%, respectively. These results indicated that S-ISL suppressed intracellular ROS production of Tca8113 cells.

### 3.3. Effects of S-ISL on Cell Cycle in Tca8113 Cells

Compared to the vehicle control, S-ISL (6.25~12.5 *μ*g/mL) induced cell accumulation in S phase with a corresponding decrease in G_2_ phase while S-ISL 25~50 *μ*g/mL markedly induced cell accumulation in G_1_ phase with a corresponding decrease in S phase (Figure 2(b)). Cell cycle studies indicated a biphasic effect of S-ISL, with a lower-concentration accumulation of the cells in the S phase and a higher-concentration in G_1_ phase.

### 3.4. Effects of S-ISL on Apoptosis in Tca8113 Cells

As shown in Figure 2(b), a fraction of cells with hypodiploid DNA content representing apoptosis can be detected using cell cycle analysis. S-ISL (25~50 *μ*g/mL) increased the percentage of sub-G_1_ DNA content in Tca8113 cells as compared with the vehicle treated groups.

**Figure 3:**
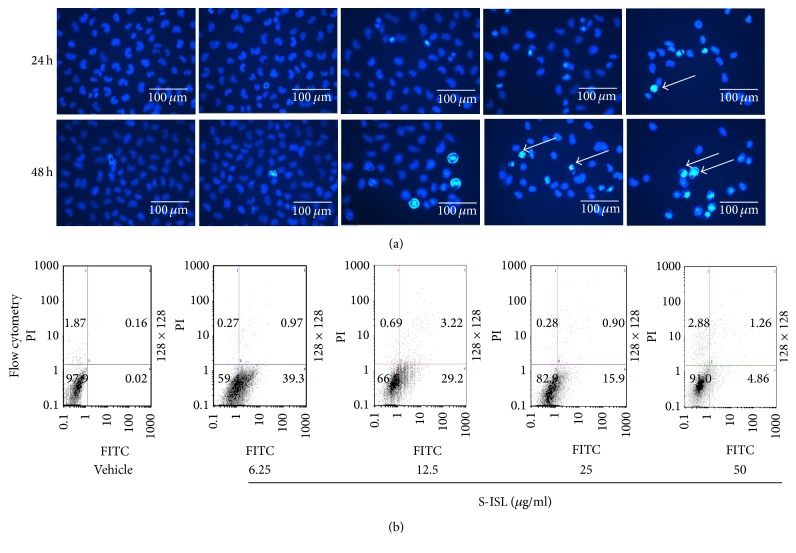
Effects of S-ISL on apoptosis: The effects of S-ISL (6.25~50 *μ*g/mL) on Tca8113 cell apoptosis were examined using fluorescence microscopy technique (a) and FCM analysis (b). Apoptosis cells with powerful fluorescence bodies of nuclear fragmentation were marked by arrows. S-ISL: synthetic isoliquiritigenin; FCM: flow cytometry.

Further, apoptotic cells were identified by chromatin morphology using DAPI. After 24 h and 48 h treatment, S-ISL (6.25–50 *μ*g/mL) induced chromatin condensation in Tca8113 cells compared to the vehicle cells (Figure 3(a)). The ratio of apoptotic cells to total cells was 0.2%, 6.2%, 10.4%, and 16.3%, respectively, after 24 h treatment of S-ISL, and the ratio for 48 h treatment was 1.6%, 12.3%, 14.6%, and 30.4%, respectively. The morphological characteristics of the vehicle cells demonstrated good spreading and flattening with no DAPI staining of nuclei (Figure 3(a)). On the other hand, Tca8113 cells pretreated with S-ISL (6.25~50 *μ*g/mL) for 24 h and 48 h displayed cell rounding, weak spreading, shrinking, and retracting of cellular processes. S-ISL treatment resulted in brighter stained nuclei of Tca8113 cells with condensed chromatin forming crescent-shaped profiles around the periphery of the nuclei.

In addition, the apoptosis-inducing effect of S-ISL was evaluated by double staining with Annexin V-FITC/PI to distinguish between living cells, early and late apoptotic cells, and necrotic cells. As shown in Figure 3(b), when Tca8113 cells were treated without S-ISL, 97.9% of cells were in a normal condition. After the cells were incubated with S-ISL (6.25~50 *μ*g/mL) for 24 h, 39.3%, 29.2%, 15.9%, and 4.86% of cells were in early phase of apoptosis whereas there were few cells in the late apoptotic/necrotic stage. These results indicated that S-ISL had a negative concentration-dependent effect on Tca8113 cell apoptosis induction, especially in the early phase of apoptosis.

### 3.5. Changes of Bax and Bcl-2 Protein Expressions in Response to S-ISL Treatment

**Figure 4:**
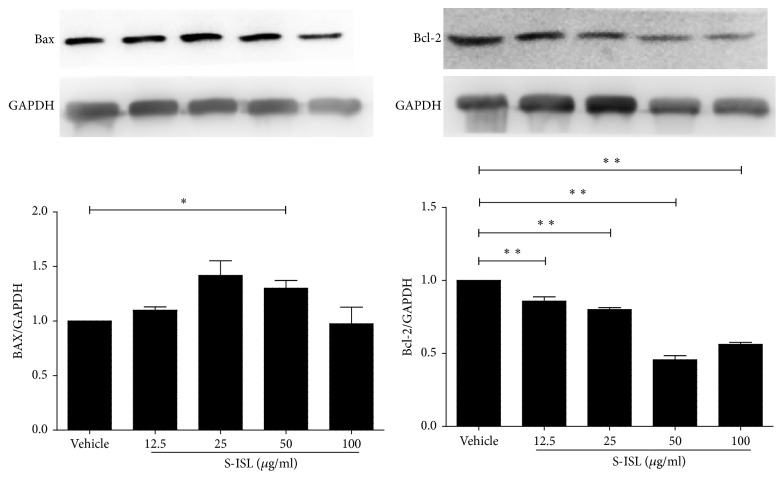
Bax and Bcl-2 protein expression in response to S-ISL: Western blots illustrate protein abundance of Bax and Bcl-2 using representative samples from each group above the graph. Graphs show Bax and Bcl-2 protein abundance normalized into GAPDH after S-ISL (12.5~100 *μ*g/mL) treatment. S-ISL: synthetic isoliquiritigenin. Values are Mean (SD). ^*∗*^*P* < 0.05, ^*∗∗*^*P* < 0.01 versus the vehicle group.

Western blotting assay was performed to evaluate the change of apoptosis regulators (Bax and Bcl-2) in response to S-ISL treatment (Figure 4). Relative to the vehicle group, S-ISL incubation from 12.5~100 *μ*g/mL significantly decreased Bcl-2 protein expression and increased Bax protein expression in Tca8113 cells.

### 3.6. Effects of S-ISL on Tca8113 Cells Adhesion, Migration, and Invasion

**Figure 5:**
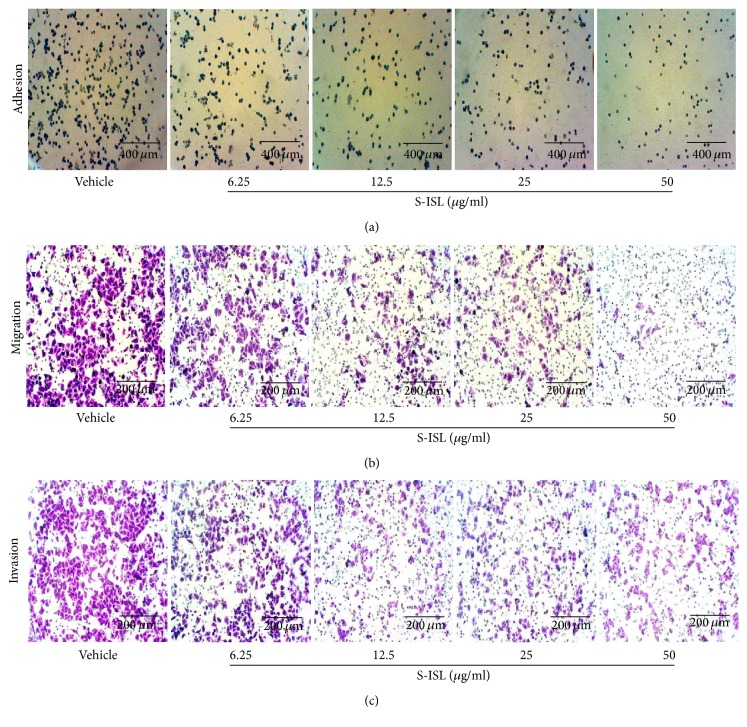
Effects of S-ISL on adhesion, migration, and invasion: The representative images were shown in regard to adhesion (a), migration (b), and invasion (c) of Tca8113 cells in response to S-ISL (6.25~50 *μ*g/mL) treatment using an inverted microscope. S-ISL: synthetic isoliquiritigenin.

Cell-matrix adhesion assay showed that S-ISL treatment remarkably decreased adhesion abilities of Tca8113 cells on matrigel-coated surface. As shown in Figure 5(a), when the concentrations of S-ISL varied from 6.25 *μ*g/mL to 50 *μ*g/mL, the inhibitory adhesion rates were 39.18%, 51.55%, 54.64%, and 64.95%, respectively (*P* < 0.05 compared with the control group).

Further, the effect of S-ISL on the migration of Tca8113 cells was examined using Boyden chamber assay. As shown in Figure 5(b), the number of Tca8113 cells passing through the polycarbonate membrane in S-ISL pretreated groups was significantly less than that in the vehicle group (*P* < 0.05). The inhibitory migration rates of S-ISL from 6.25 *μ*g/mL to 50 *μ*g/mL were 21.33%, 58.59%, 62.73%, and 79.30%, respectively.

As shown in Figure 5(c), S-ISL from 6.25 *μ*g/mL to 50 *μ*g/mL also significantly inhibited Tca8113 cells invasion compared to the vehicle group (*P* < 0.05) and the inhibitory rates were 28.29%, 51.05%, 51.33%, and 58.04%, respectively. These results indicated that S-ISL inhibited the adhesion, migration, and invasion abilities of Tca8113 cells in a concentration-dependent manner.

### 3.7. Changes of MMP-2 and MMP-9 Activities in Response to S-ISL

**Figure 6:**
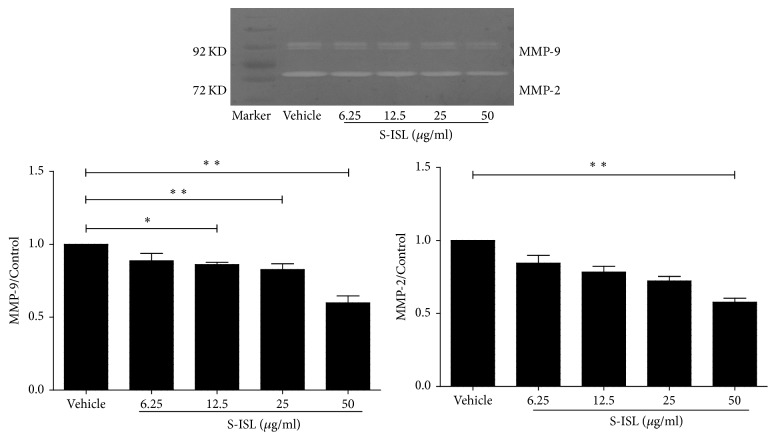
MMP-2 and MMP-9 activities in response to S-ISL: MMP-2 and MMP-9 proteolytic activities were measured in Tca8113 cells pretreated with S-ISL (6.25~100 *μ*g/mL) at various concentrations for 24 h using Gelatin zymography assay. S-ISL: synthetic isoliquiritigenin; MMP: matrix metalloproteinase. Values are Mean (SD). ^*∗*^*P* < 0.05, ^*∗∗*^*P* < 0.01 versus the vehicle group.

In order to explore the possible antimetastatic mechanism of S-ISL in Tca8113 cells, the activities of MMP-2 and MMP-9 were tested using Gelatin zymography assay. S-ISL treatment (6.25~50 *μ*g/mL) showed a concentration-dependent reduction in MMP-2 and MMP-9 activities. The inhibitory rates of MMP-2 were 13.57%, 21.02%, 24.45%, and 38.09%, and those of MMP-9 were 13.03%, 14.01%, 17.53%, 44.76%, respectively (Figure 6). These results indicate that the inhibition of MMP-2 and MMP-9 by S-ISL might play a key role in invasion and metastasis of Tca8113 cells.

## 4. Discussion

Our study shows that S-ISL has multiple anticancer effects on human tongue squamous carcinoma cells, including specific and biphasic effects of inhibiting proliferation, inducing cell apoptosis and impeding adhesion, migration, and invasion. The anticancer effects are attributed to increased levels of apoptotic Bax/Bcl-2 ratio and decreased activity of MMP-2 and MMP-9, which is likely to be mediated via antioxidant mechanisms of S-ISL.

The intracellular level of ROS is generally elevated in cancer cells, affecting all characteristics of cell behaviour, including cell cycle progression and proliferation, cell survival, and apoptosis and metastasis [26]. Like its natural counterpart, our data showed that S-ISL also had the same antioxidant capacity, providing the rational basis for our ongoing investigation of its antitumor effects.

Firstly we observed the effects of S-ISL on the proliferation of human tongue squamous carcinoma line Tca8113, in comparison with human liver carcinoma HepG2 cells and rat pheochromocytoma PC12 cells. S-ISL inhibited the growth of Tca8113, HepG2, and PC12 cells in a concentration- and time-dependent manner. Amongst these cells, Tca8113 cells exhibited the strongest response to S-ISL treatment. The inhibitory concentration of S-ISL observed was similar to the reports using the natural ISL [10, 16]. These results suggest that the effect of S-ISL against Tca8113 cells is specific and provide a first glimpse of the types of cancer cells that may benefit from such treatment. Further we revealed that the inhibitory effect of S-ISL on Tca8113 growth was due to a biphasic effect of S-ISL on Tca8113 cell cycle with a lower-concentration accumulation in the S phase and a higher-concentration in G_1_ phase. Such effect is apparently different from G_2_/M phase arrest induced by ISL in human lung cancer cell line A549 and human oral squamous cell carcinoma [17, 27], confirming the specific effects of S-ISL on Tca8113 cells.

Under physiological development, cell proliferation and apoptosis maintain proper balance. Compelling evidence indicates that some oncogenic mutations disrupt apoptosis, leading to tumor initiation, progression, or metastasis [28]. To test whether S-ISL could induce tumor cells apoptosis, we employed a variety of techniques including flow cytometry, DAPI staining, and double staining with Annexin V-FITC/PI to observe cell apoptosis related changes. Our data showed that S-ISL significantly increased the number of dead cells in a dose- and time-dependent manner. However, the dose dependent effects from both assays of DAPI staining and Annexin V-FITC/PI are contradictory in that the low dose of S-ISL showed strongest apoptosis-inducing effect using Annexin V-FITC/PI method while DAPI staining demonstrated a positive concentration-dependent effect of S-ISL on Tca8113 cell apoptosis induction. We argue that the apparent discrepancy is possibly attributed to different phases/aspects of apoptosis examined and different regulatory mechanisms of ISL in different concentrations. Annexin V-FITC/PI is used to detect early phase of apoptosis by probing phosphatidylserine translocated from the internal part of the plasma membrane to the external portion of the membrane. In contrast, DAPI staining is used to examine the morphological changes to quantify the apoptotic cells which are likely to happen in the late phase of apoptosis. Thus the same concentration of S-ISL may have different effects if examined in different phases of the apoptotic process. Additionally, we cannot exclude the possibility that different cell signalling pathways may be initiated to regulate the cell death under different concentrations of ISL exposure, which need further studies. In analyzing morphological characteristics, Tca8113 cells pretreated with S-ISL had typical changes including cell rounding, reduced spreading, shrinking and retracting of cellular processes, and brighter stained nuclei with condensed chromatin forming crescent-shaped profiles around the periphery of the nuclei. All of these changes indicated that S-ISL caused apoptosis of Tca8113 cells. One of the major genes that regulate apoptosis is the Bcl-2 family, which plays a critical role in the mitochondrial pathway of apoptosis as either promoters (e.g., Bax) or inhibitors (e.g., Bcl-2) of the cell death process [29]. Therefore, the alteration of intracellular Bax and Bcl-2 expression ratio can affect mitochondrial content release [30] and determine susceptibility to apoptosis [31]. In agreement with the previous studies [10, 32], Tca8113 cells with S-ISL treatment had an increase of Bax and a decrease of Bcl-2 protein expression and alteration of proapoptotic Bax/antiapoptotic Bcl-2. We therefore conclude that the mechanism that S-ISL promoted apoptosis may be via changing the levels of the Bcl-2 family and the ratio of Bax and Bcl-2.

The majority of cancer related deaths are caused by metastases. Therefore, it is important to develop therapeutic interventions specifically targeting the metastatic process. The metastatic cascade includes a succession of six distinct steps: localized invasion, intravasation, translocation, extravasation, micrometastasis, and colonization [33]. The basic strategy of our interventions is aimed at disturbing cancer cells' adhesion, migration, and invasion abilities which are basic steps of metastasis. In this study, S-ISL inhibited Tca8113 cells adhesion, migration, and invasion abilities, indicating that S-ISL is a potential antimetastasis drug. MMP is regarded as a key player of tumor invasion and metastasis. The proteolytic activity of MMP is able to degrade extracellular matrix (ECM) proteins and subsequently induces or enhances tumor survival, invasion, and metastasis [34]. ROS in cancer not only regulate the expression of MMPs, but also inactivate their inhibitors TIMP (tissue inhibitor of metalloproteinase) [26]. Indeed, Tca8113 cells treated with S-ISL with antioxidant characteristic showed a concentration-dependent reduction of MMP-2 and MMP-9 activities. Thus anti-invasion and metastasis effects of S-ISL on Tca8113 are likely to be achieved via inhibiting MMP activities.

Finally S-ISL showed a concentration-dependent manner in promotion of apoptosis, inhibiting proliferation, adhesion, migration, and invasion of Tca8113 cells, with an effective and optimal concentration from 25 to 50 *μ*g/mL. Previously, Lee and colleagues reported that plasma ISL concentration could reach 4.16 ± 1.80 mg/mL after 30 min of administration of a 50 mg/kg intravenous dose of ISL to rats [35]. A recent study showed that intraperitoneal administration of 1 mg/kg of ISL significantly decreased tumor size and inhibited the viability of cancer cells in xenograft mouse model without apparent side effects on normal cells [36]. Therefore the concentrations of S-ISL used in this study are highly achievable in vivo, further supporting its promise in clinical application.

## 5. Conclusions

In summary, our data showed that S-ISL had antiproliferative, proapoptotic, and antimetastatic effects on human tongue squamous carcinoma cells through its antioxidant mechanism and potentially could be a therapeutic agent against tongue cancer.

## Supplementary Material

Figure a. The molecular structure of synthesis Isoliquiritigenin (S-ISL). b. Carbon spectra of S-ISL. c. Hydrogen spectra of S-ISL. d. Spectrum of S-ISL. e. High Performance Liquid Chromatography (HPLC) of S-ISL

## Abbreviations

TSCC: Squamous cell carcinoma of the tongue
ISL: Isoliquiritigenin
ROS: Reactive oxygen species
S-ISL: Synthesis isoliquiritigenin
HPLC: High performance of liquid chromatography
DMEM: Dulbecco's modified Eagle's medium
DMSO: Dimethylsulfoxide
DCFH-DA: Dichlorodihydrofluorescein diacetate
SRB: Sulforhodamine B
FCM: Flow cytometry
PBS: Phosphate-buffered saline
SDS: Sodium dodecyl sulphate
MMP: Matrix metalloproteinase
ANOVA: Analysis of variance
ECM: Extracellular matrix
TIMP: Tissue inhibitor of metalloproteinase.
