# Supplementary material for: Synthetic Isoliquiritigenin Inhibits Human Tongue Squamous Carcinoma Cells through Its Antioxidant Mechanism
Source: Oxid Med Cell Longev. 2017 Jan 22;2017:1379430. doi: 10.1155/2017/1379430 (PMC5292127; doi:10.1155/2017/1379430)
Supplement: Supplementary file 1 — Figure a. The molecular structure of synthesis Isoliquiritigenin (S-ISL). b. Carbon spectra of S-ISL. c. Hydrogen spectra of S-ISL. d. Spectrum of S-ISL. e. High Performance Liquid Chromatography (HPLC) of S-ISL [file 1379430.f1.pptx]

## Slide 1
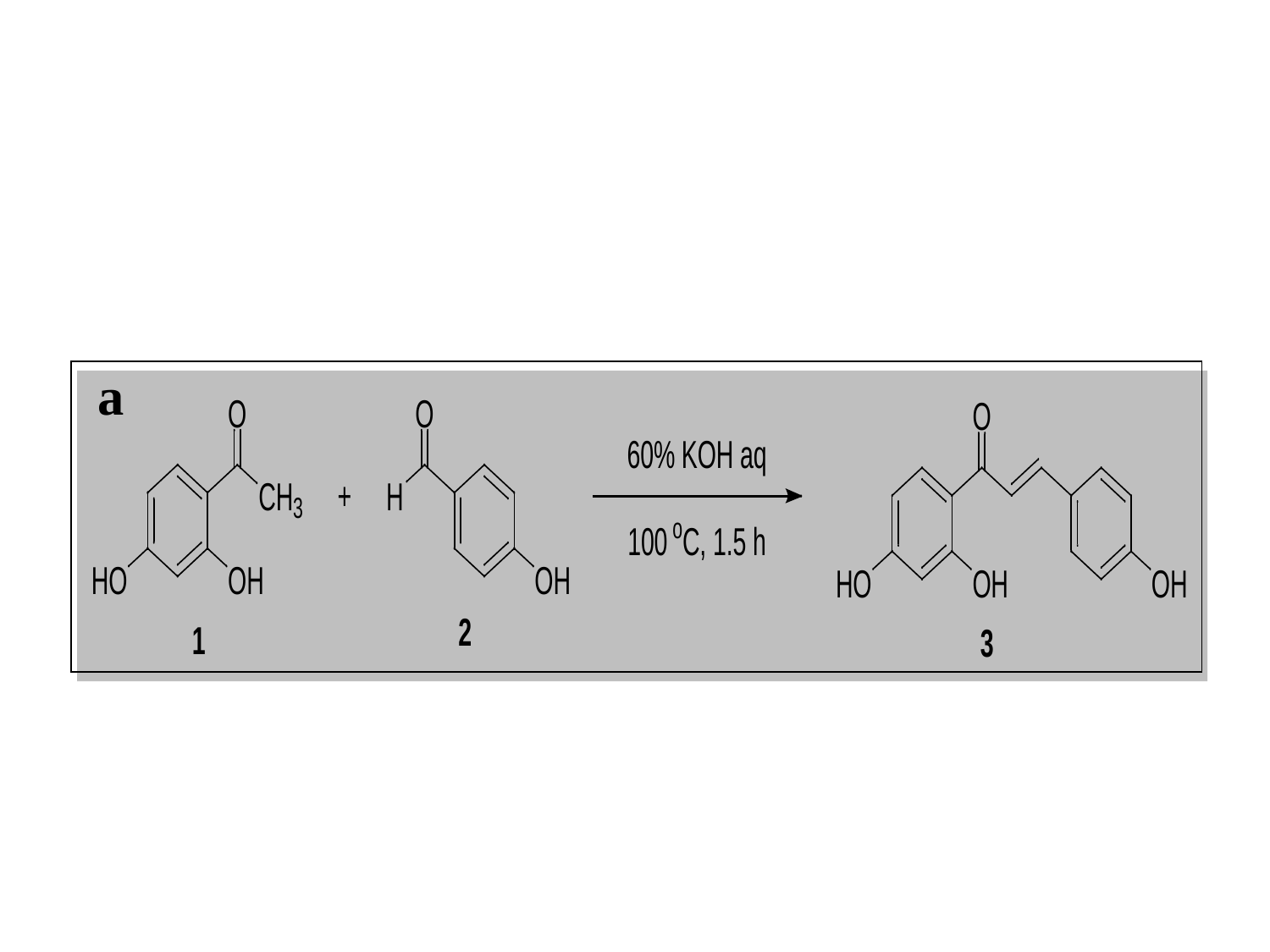

a

## Slide 2
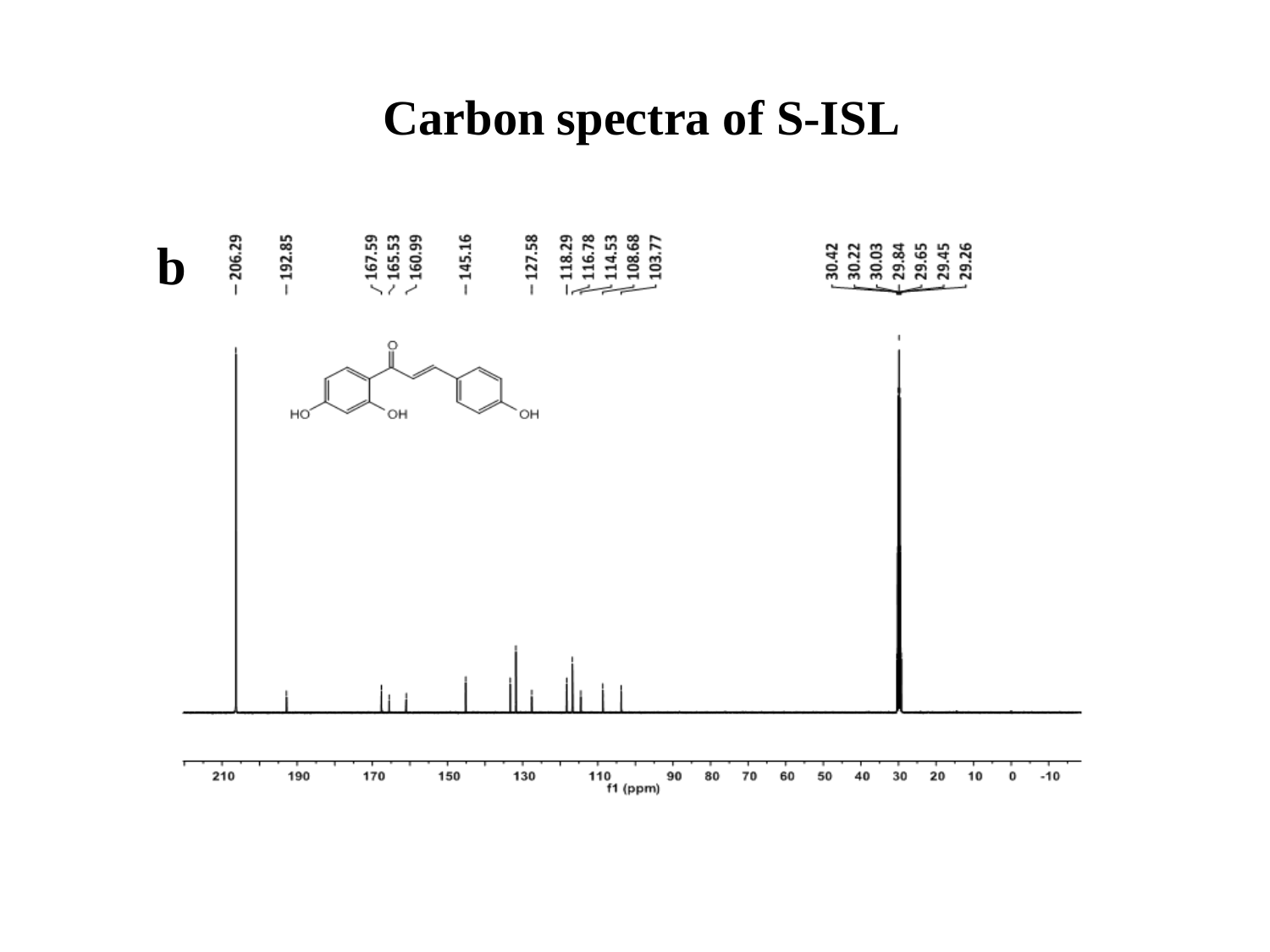

Carbon spectra of S-ISL
b

## Slide 3
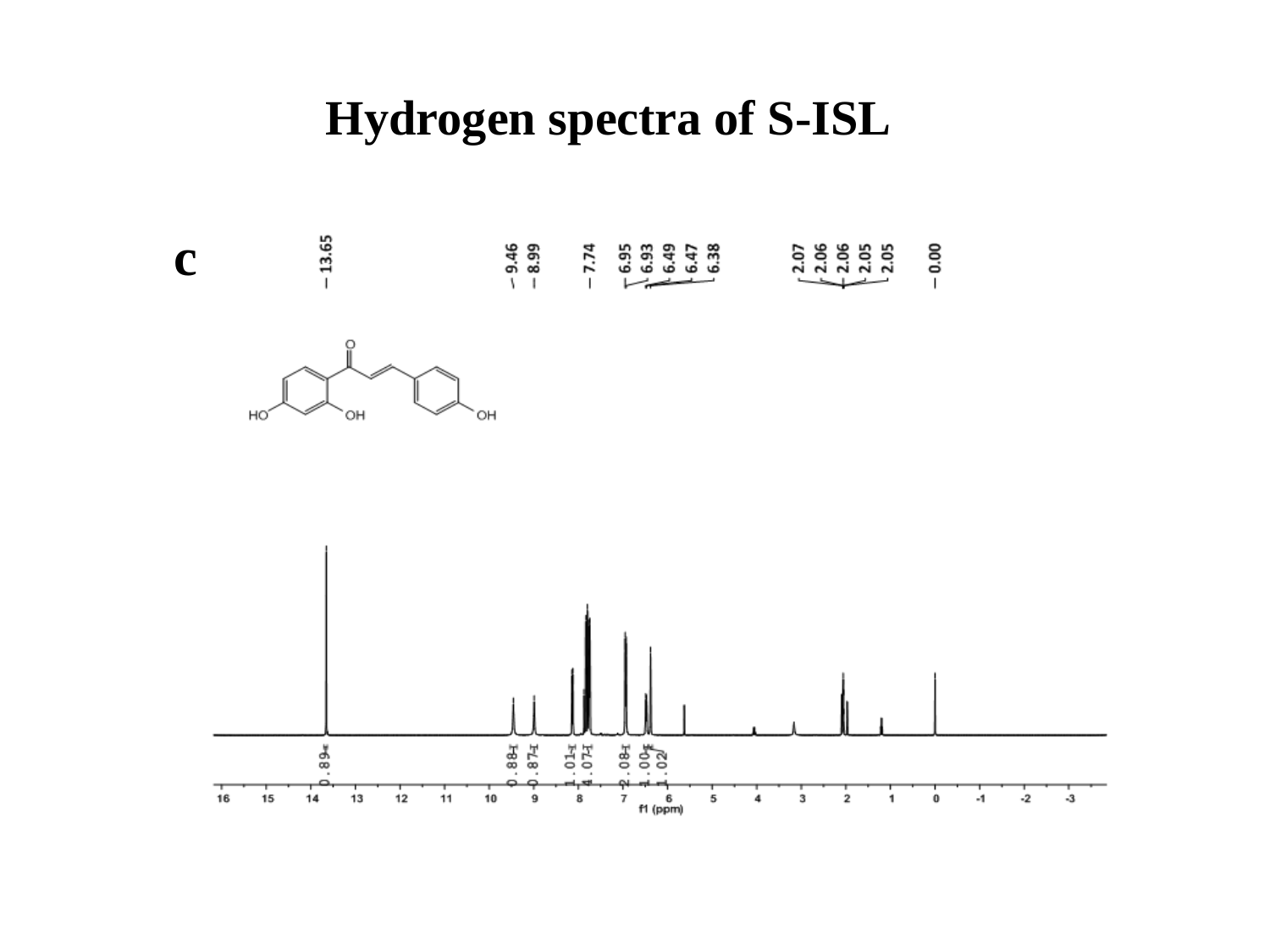

Hydrogen spectra of S-ISL
 c

## Slide 4
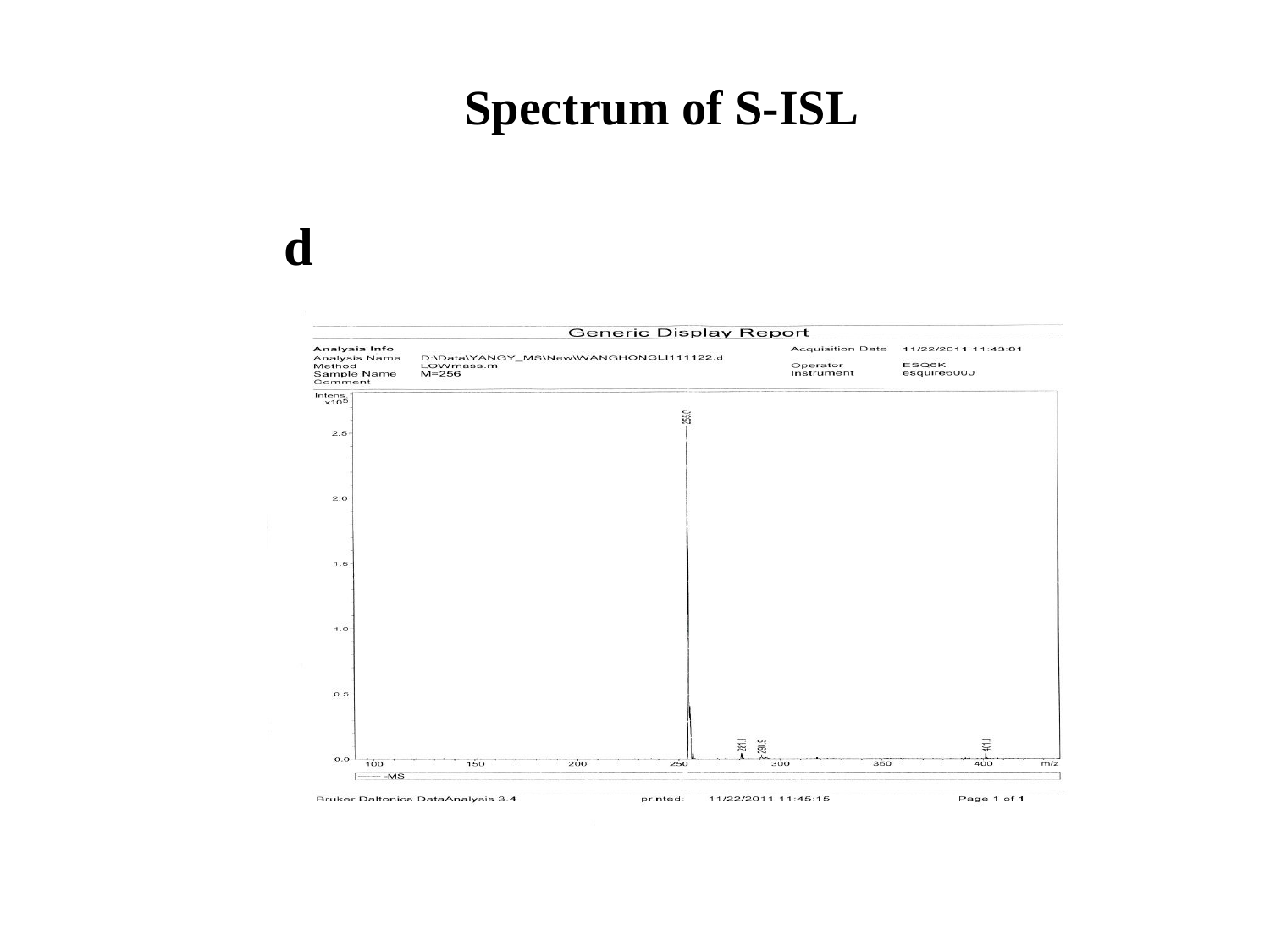

Spectrum of S-ISL
 d

## Slide 5
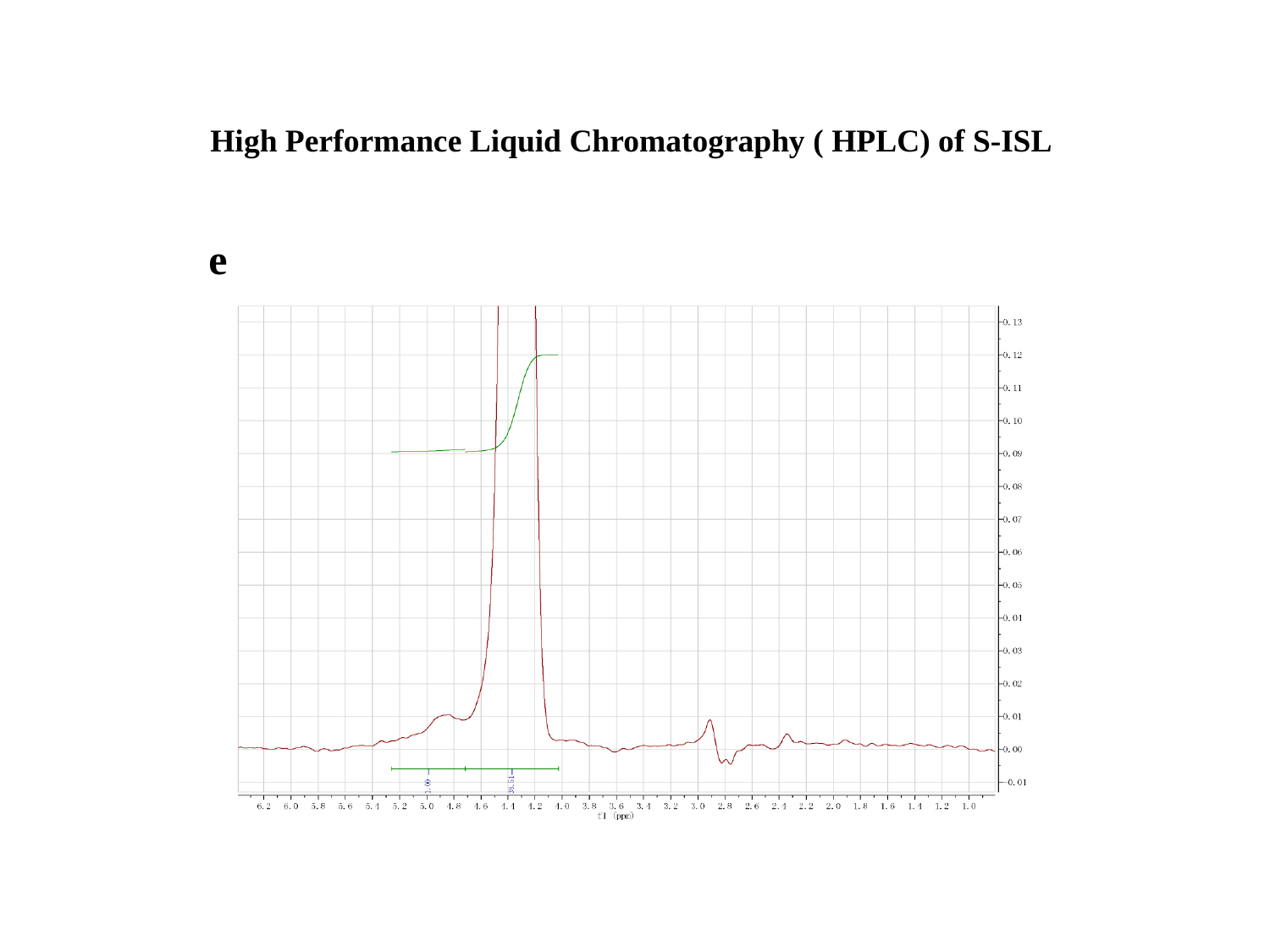

High Performance Liquid Chromatography ( HPLC) of S-ISL
 e
